# From superstition to evidence-based information: evaluation of websites about alcohol available in Portuguese

**DOI:** 10.1186/1940-0640-8-S1-A23

**Published:** 2013-09-04

**Authors:** Leonardo Fernandes Martins, Henrique Pinto Gomide, Telmo Mota Ronzani

**Affiliations:** 1 Federal University of Juiz de Fora, Juiz de Fora, Minas Gerais, Brazil

## 

Among several strategies for reducing alcohol consumption, internet-based interventions present good indicators of cost-effectiveness. The internet, in Brazil, has approximately 76 million users, which consists of 37.8% of the entire population. The purpose of this study was to evaluate websites and identify interventions aimed at internet-assisted reduction for alcohol consumption and alcoholism available in Portuguese. In order to evaluate the websites about alcohol available on the internet, a survey was conducted on Google Adwords to define which were the words most used by internet users. The searches were conducted through the three most used search engines in Brazil. First page results of each search were collected and classified by two researchers through an adapted websites rating scale. One hundred and eighty-five websites were found. Duplicate records were removed. Of the 69 websites analyzed, 31.9% lacked information on alcohol-related content, 26.1% included articles on the subject, but not so structured for alcohol users, 24.6% provided direct information on the consumption reduction processes or alcohol withdrawal, and the remaining 17.4% were classified as selling products, links to other websites, and product and facilities advertising for alcoholism treatment. Of the 17 sites that provided information about the consumption reduction process or abstinence, 9 were classified as evidence-based content and 8 as superstitions or beliefs like popular teas and potions. Most of the internet content is not evidence-based. Thus, it is necessary to develop websites with appropriate information for users who wish to reduce their alcohol consumption or for those who need to be referred to public health professionals.

## Funding

UFJF, FAPEMIG, CNPq.

